# Association between oxidative balance score and rheumatoid arthritis in female: a cross-sectional study

**DOI:** 10.1186/s12905-024-03066-3

**Published:** 2024-04-06

**Authors:** Rui La, Liyu Zhou, Yunfei Yin, Lingchen Lu, Lisong Li, Dinghua Jiang, Lixin Huang, Qian Wu

**Affiliations:** 1 Department of Orthopedic Surgery and Sports Medicine, Institute of Orthopedics, The First Affiliated Hospital of Soochow University, Soochow University, Jiangsu, China; 2 Department of Cardiology, The First Affiliated Hospital of Soochow University, Jiangsu, China; 3 Department of Pediatric Surgery, Maternal and Child Health Care Hospital of Kunshan, Jiangsu, China; 4 Research Institute of Clinical Medicine, Department of Orthopedic Surgery and Biochemistry, Jeonbuk National University Medical School, Jeonju, Republic of Korea

**Keywords:** Oxidative balance score, Rheumatoid arthritis, National health and nutrition examination survey, Cross-sectional study

## Abstract

**Objective:**

Although oxidative stress is a recognized factor of inflammation, the correlation between oxidative balance score (OBS), a biomarker indicating the balance of oxidation and antioxidant, and rheumatoid arthritis (RA), an immune system disease that tends to occur in women, remains unexplored. Hence, the aim of this study was to investigate the potential association between OBS and RA in women.

**Methods:**

Observational surveys were performed by employing information extracted from the National Health and Nutrition Examination Survey (NHANES) for the period 2007–2018. Various statistical techniques were employed to investigate the association between OBS and RA, encompassing multivariable logistic regression analysis, subgroup analyses, smooth curve fitting, and threshold effect analysis.

**Results:**

The study included 8219 female participants, including 597 patients with RA. The results showed that higher Total OBS (TOBS) significantly correlated with lower RA prevalence in the entirely modified model [odd ratio (OR) = 0.968; 95% confidence interval (CI) = 0.952 to 0.984; *P* = 0.0001]. Dietary OBS (DOBS) and lifestyle OBS (LOBS) also negatively correlated with RA. This association was remarkably consistent across TOBS subgroups by age, race, education level, family poverty-to-income ratio (PIR), hypertension and diabetes. Smooth curve fitting and threshold effect analysis also revealed the linear relationship between OBS and RA.

**Conclusions:**

Overall, OBS was negatively associated with RA in female. This study suggested that an antioxidant diet and lifestyle may be promising measures to prevent RA in female.

**Supplementary Information:**

The online version contains supplementary material available at 10.1186/s12905-024-03066-3.

## Introduction

Rheumatoid arthritis (RA) is an autoimmune disease that symmetrically affects numerous joints, predominantly the hand and foot joints [1]. As RA progresses, patients become increasingly intolerant of joint pain and experience loss of joint mobility, joint deformation and even disability, severely diminishing the quality of life [2]. According to the Global Burden of Diseases, Injuries, and Risk Factors Study, an estimated 17.6 million people worldwide were affected by RA. Remarkably, women were disproportionately affected, with their prevalence being roughly 2.45 times higher than men post age-standardization [3]. Unfortunately, current therapeutic approaches cannot cure RA, patients can only be given non-steroidal anti-inflammatory drugs and disease-modifying anti-rheumatic drugs to mitigate symptoms and slow the progression of RA [4]. Advanced cases with severe joint stiffness and dislocation might necessitate joint replacement surgery, which may require multiple operations. Clearly, addressing the challenges of RA remains a pressing issue.

Notably, oxidative stress has been identified as a pivotal factor in the pathology of RA [5]. Reactive oxygen species (ROS) are generated in the course of RA immune response [6], among which hydrogen peroxide inhibits proteoglycan synthesis and hydroxyl radical accelerates proteoglycan degradation [7]. In addition, hydroxyl radicals can interact with chloride ions to produce hypochlorous acid [5], which also restrains proteoglycan synthesis by activating matrix metalloproteinases, thus mediating cartilage repair disorders and cartilage dysfunction [7, 8]. In recent years, a growing number of studies emphasized the effects of antioxidants and pro-oxidants on RA, with daily diet and lifestyle taking center stage. Fruits and vegetables rich in vitamins and polyphenol compounds, as well as olive oil rich in omega-3 fatty acids and tocopherols, are known for their antioxidant nature, removing or inhibiting free radicals, and can significantly reduce the risk of RA [9, 10]. On the contrary, smoking, recognized for its pro-oxidative attribute due to releasing free radicals [6], significantly increases the risk of RA either actively or passively [11, 12]. Additionally, fat accumulation is also closely related to oxidative stress, and immoderate caloric intake induces ROS production [13]. Hence, Obesity is associated with an elevated risk of developing RA [14, 15].

The oxidative balance score (OBS) serves as a novel scoring system to evaluate the antioxidant/oxidative level, which represents the comprehensive impact of 20 factors on the bodily oxidative stress state. Higher OBS indicates elevated body antioxidant levels. Previous studies have shown that OBS is inversely associated with multiple diseases, such as neuropsychiatric diseases, metabolic diseases and cardiovascular diseases. Individuals with higher OBS exhibited fewer depressive symptoms [16], increased femur bone density, and reduced osteoporosis risk in postmenopausal women [17]. Lifestyle components like weight management, balanced diet, and regular exercise, are all known antioxidants, leading to high OBS and decreasing diabetes risk [18]. Subsequent research from the same team revealed a negative correlation between OBS and metabolic syndrome [19], further verifying the potential protective role of OBS against metabolic diseases. Besides, there existed a negative link between OBS and ischemic heart disease, with each OBS unit increasing reducing the risk by 18% [20]. According to the protective effect of high OBS on many diseases, we speculate that OBS [including Total OBS (TOBS), Dietary OBS (DOBS) and lifestyle OBS (LOBS)] may also exhibit a negative correlation with RA in female population. And National Health and Nutrition Examination Survey (NHANES) data as well as multiple statistical techniques were utilized to test this hypothesis.

## Methods

### Survey description

NHANES is a comprehensive cross-sectional study conducted by the National Center for Health Statistics to assess the health and nutritional status of the U.S. population. This study amasses demographic data, dietary data, examination data, and laboratory data from both adults and children, and uses questionnaires to collect information about the diseases, medical conditions, and health indicators, which are accessible without charge on their website. Notably, these data offer insights into protective and risk factors for various diseases and to guide public health strategies.

### Study population

**Fig. 1:**
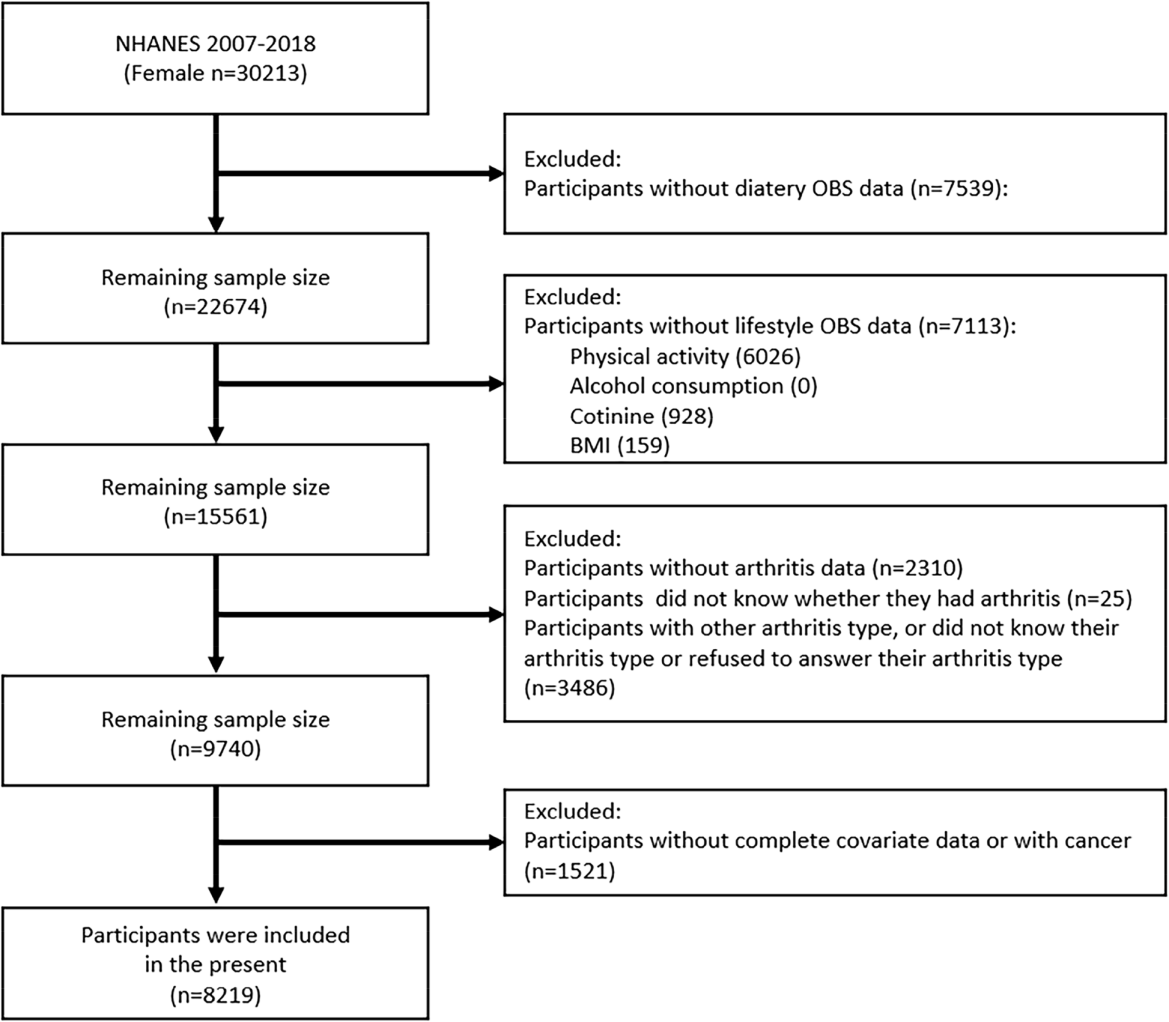
Flowchart of the participant selection from NHANES 2007–2018

This study included 30,213 female participants across six 2-year cycles: 2007–2018. These respondents were surveyed for DOBS composition and LOBS composition data. Initially, 7539 participants were excluded due to incomplete DOBS data. Subsequently, 7113 participants were excluded owing to a lack of LOBS data, including physical activity, cotinine, and BMI. The next elimination involved 2310 participants absent from the arthritis questionnaire and 25 participants who were uncertain about their arthritis condition. Another 3486 participants were excluded due to other types of arthritis, uncertainties or refusals to specify their arthritis type. Furthermore, participants lacking complete covariate data or with cancer were also excluded, amounting to a total of 1521 participants. After all considerations, a total of 8219 participants were recruited in our study (Fig. 1).

### Assessment of OBS (exposure)

OBS can be bifurcated into DOBS and LOBS. The former encompasses 16 components: dietary fiber, carotene, riboflavin, niacin, total folate, vitamin B6, vitamin B12, vitamin C, vitamin E, calcium, magnesium, zinc, copper, selenium, iron, and total fat. Iron and fat exhibit pro-oxidative properties, whereas the rest are antioxidants. It should be noted that the average data derived from the first-day dietary interview (collected in-person in the Mobile Examination Center) and the second-day dietary interview (collected by telephone) were used as the outcome. Also, the sum of α-carotene and β-carotene was labeled as total carotene in the present study.

LOBS is composed of physical activity, alcohol consumption, smoking, and body mass index (BMI). Physical activity, an antioxidant factor, was assessed by the physical activity questionnaire, which highlighted five principal activities: vigorous work activity, moderate work activity, walking or cycling, vigorous recreational activity, and moderate recreational activity. The levels of physical activity were assessed according to the following formula: metabolic equivalent (MET) score × frequency per week × activity duration, with 600 MET-min/week and 3000 MET-min/week as thresholds for low, medium and high levels of physical activity [21]. Notably, the MET scores for the five activities listed above were 8, 4, 4, 8, and 4, in that order, in accordance with the NHANES recommendations [22]. Conversely, alcohol consumption, cotinine and BMI were pro-oxidative factors. Participants were segmented into non-drinkers (0 g/day), non-alcoholics (0–15 g/day), and alcoholics (≥ 15 g/day) based on their daily alcohol consumption. In particular, the categorization of alcohol consumption referred to a preceding NHANES study on OBS, where a scoring system that assigns scores from 2 to 0 in descending order was adopted [23]. Smoking behaviors were evaluated by serum cotinine levels. This methodology aligned with a prior study [23]. Serum cotinine, as the principal nicotine metabolite, possesses a more stable half-life, thereby providing an objective indicator of both active and passive tobacco exposure. Consequently, it serves as a reliable and sensitive marker for tobacco exposure at this juncture [24]. In addition, participants were defined as normal or lean (BMI < 25.000 kg/m^2^), overweight (25.000 kg/m^2^ ≤ BMI < 30.000 kg/m^2^), and obese (BMI ≥ 30.000 kg/m^2^) adhering to the World Health Organization criteria.

Except for physical activity, alcohol consumption and BMI, each item was grouped according to a weighted tertile, denoted as groups 1, 2, and 3 from lowest to highest. Antioxidant factors were assigned to 0–2 points, and pro-oxidation factors were assigned to 2 − 0 points (Table 1). Additionally, the original scores of 20 items of TOBS for RA and Non-RA were also displayed in Supplemental Table 1.

**Table 1:** OBS components

OBS components	Property	0	1	2
DOBS components				
Dietary fiber (g/d)	A	< 11.3	11.3–17.4	≥ 17.4
Carotene (mcg/d)	A	< 688.5	688.5-2488.147	≥ 2488.147
Riboflavin (mg/d)	A	< 1.3595	1.3595–1.96025	≥ 1.96025
Niacin (mg/d)	A	< 16.59475	16.59475–23.6026	≥ 23.6026
Total folate (mcg/d)	A	< 261.5	261.5–389	≥ 389
Vitamin B6 (mg/d)	A	< 1.3071	1.3071–1.9224	≥ 1.9224
Vitamin B12 (mcg/d)	A	< 2.576	2.576–4.44	≥ 4.44
Vitamin C (mg/d)	A	< 40.2	40.2-90.492	≥ 90.492
Vitamin E (mg/d)	A	< 5.075	5.075-	≥ 7.985
Calcium (mg/d)	A	< 624.083	624.083	≥ 937
Magnesium (mg/d)	A	< 208.5	208.5–290	≥ 290
Zinc (mg/d)	A	< 7.21	7.21-10.319	≥ 10.319
Copper (mg/d)	A	< 0.851	0.851–1.2024	≥ 1.2024
Selenium (mcg/d)	A	< 76.35	76.35-108.292	≥ 108.292
Iron (mg/d)	P	≥ 14.015	9.776–14.015	< 9.776
Total fat (g/d)	P	≥ 76.465	56.861–76.465	< 56.861
LOBS components				
Physical activity (MET-minute/week)	A	< 600	600–3000	≥ 3000
Alcohol (g/d)	P	≥ 15	0–15	0
Body mass index (kg/m2)	P	≥ 30.000	25.000–30.000	< 25.000
Cotinine (ng/mL)	P	≥ 0.096	0.015–0.096	< 0.015

A for the antioxidant, P for the pro-oxidant

### Assessment of the diagnosis of RA (outcome)

The diagnosis of RA was obtained through the medical conditions questionnaire in the NHANES database, and participants were required to answer the following questions. The first is “Doctor ever said you had arthritis”. If the answer was “Yes”, the participant was asked the next question, “Which type of arthritis?”, to report the type of arthritis they had, including RA, osteoarthritis, and other types of arthritis. The approach of utilizing self-reported questionnaires for RA diagnosis aligned with methodologies employed in both our previous research and other studies using NHANES data on RA [25–27]. Moreover, evidence suggested that self-reported RA diagnosis can be deemed reliable in extensive studies where a direct examination by a rheumatologist is impractical [28].

### Covariate definitions

To adjust for the effect of confounding factors in this study, the following items were included in the adjusted covariate assessment: age, race, education level, poverty-to-income ratio (PIR), energy intake, hypertension and diabetes. Hypertension and diabetes diagnoses were also obtained from the NHAENS questionnaire, with the former containing “Yes” and “No” as responses, and the latter containing “Yes”, “No”, and “Borderline” as answers, on the basis of which the groups of the two diseases were established. Besides, the systemic immune inflammation index (SII), which was derived from platelet count multiplying neutrophil count dividing by lymphocyte count was used to reflect inflammation levels.

### Statistical analysis

The population baseline characteristics were presented as mean ± standard deviation for continuous variables and as frequencies and percentages for categorical variables. Multivariate logistic regression was used to analyze the correlation between OBS (including TOBS, DOBS and LOBS) and RA. On the one hand, whether OBS as a continuous variable has a linear relationship with RA was explored. On the other hand, OBS was categorized into four groups by quartile calculations and the median of each group was included in the logistic regression as a continuous variable to test the *P*-value for trend [29]. In the process, three models were constructed. Model 1: crude model. Model 2: adjusted for age, race, education level, PIR and energy intake. Model 3: adjusted for hypertension and diabetes based on Model 2. The associations between OBS and RA in different age, race, education level, PIR, hypertension and diabetes subgroups were also explored. Threshold effect analysis and smooth curve fitting study were performed as well [30, 31]. All statistical analyses were performed with the use of R software (version 4.2.2) and EmpowerStats (version 2.0). The significance level was set at bilateral *P* < 0.05.

## Results

### Population baseline characteristics

Baseline characteristics of 8219 female subjects were presented in Table 2. The majority were non-Hispanic white, more than half of the participants had an education level surpassing high school, and 37.13% of the participants reported a median PIR. Prevalence of RA was 7.26% in the present study. Furthermore, RA patients were significantly more elderly than controls, with lower levels of education and income. Moreover, RA patients in this study also showed a higher prevalence of hypertension and diabetes. More importantly, TOBS, DOBS and LOBS were all markedly diminished in participants with RA.

**Table 2:** Baseline characteristics of participants

	ALL	Non-RA	RA	* P*-value
	*N* = 8219	*N* = 7622	*N* = 597	
**Age (year)**				< 0.001
≤ 44	4571 (55.62%)	4483 (58.82%)	88 (14.74%)	
45–59	1989 (24.20%)	1814 (23.80%)	175 (29.31%)	
≥ 60	1659 (20.18%)	1325 (17.38%)	334 (55.95%)	
**Race**				< 0.001
Mexican American	1369 (16.66%)	1289 (16.91%)	80 (13.40%)	
Other Hispanic	926 (11.27%)	847 (11.11%)	79 (13.23%)	
Non-Hispanic White	3102 (37.74%)	2890 (37.92%)	212 (35.51%)	
Non-Hispanic Black	1804 (21.95%)	1613 (21.16%)	191 (31.99%)	
Other race	1018 (12.39%)	983 (12.90%)	35 (5.86%)	
**Education level**				< 0.001
< High school	1656 (20.15%)	1461 (19.17%)	195 (32.66%)	
High school	1685 (20.50%)	1550 (20.34%)	135 (22.61%)	
> High school	4878 (59.35%)	4611 (60.50%)	267 (44.72%)	
**PIR**				< 0.001
< 1.3	2759 (33.57%)	2480 (32.54%)	279 (46.73%)	
[1.3, 3.5)	3052 (37.13%)	2852 (37.42%)	200 (33.50%)	
≥ 3.5	2408 (29.30%)	2290 (30.04%)	118 (19.77%)	
**Hypertension**				< 0.001
Yes	2209 (26.88%)	1842 (24.17%)	367 (61.47%)	
No	6010 (73.12%)	5780 (75.83%)	230 (38.53%)	
**Diabetes**				< 0.001
Yes	709 (8.63%)	564 (7.40%)	145 (24.29%)	
No	7362 (89.57%)	6929 (90.91%)	433 (72.53%)	
Borderline	148 (1.80%)	129 (1.69%)	19 (3.18%)	
**SII**	541.66 ± 325.38	538.34 ± 312.50	583.96 ± 457.40	0.896
**TOBS**	20.41 ± 7.25	20.58 ± 7.23	18.27 ± 7.18	< 0.001
**DOBS**	16. 01 ± 6.84	16.14 ± 6.82	14.38 ± 6.93	< 0.001
**LOBS**	4.40 ± 1.56	4.44 ± 1.57	3.89 ± 1.44	< 0.001

### Association between the OBS and RA

The association between TOBS and RA was examined through multivariate logistic regression and presented in Table 3. When OBS as a continuity variable, there existed a pronounced inverse association between TOBS and RA [odd ratio (OR) = 0.968, 95% confidence interval (CI) = 0.952 to 0.984] in the model 3 with completely adjustment, indicating that TOBS may serve as a protective factor for RA. Subsequently, the TOBS was grouped by quartile, with the lowest quartile of the TOBS as the reference group. In each model, the highest quartile of TOBS showed a pronounced negative association with RA, and this downtrend was statistically evident (*P* for trend < 0.001). In core model 3, the OR (95%CI) of group Q4 (26 ≤ OBS ≤ 38) was 0.546 (0.393 to 0.760), the TOBS was strongly negatively correlated with RA in group Q4 (*P* = 0.0003), which implies that after fully considering the influence of confounding factors, the probability of RA decreased by 45.4% for every 1 point increase in OBS within this interval (26 ≤ OBS ≤ 38).

**Table 3:** The relationship between TOBS and RA

	**OR (95% CI)**, ***P*-value**			
	**Model 1**^**a**^	**Model 2**^**b**^	**Model 3**^**c**^	
	RA			
TOBS	0.956 (0.945, 0.968), < 0.0001	0.966 (0.950, 0.982), < 0.0001	0.968 (0.952, 0.984), 0.0001	
Q1(4–13)	Reference	Reference	Reference	
Q2(14–20)	0.734 (0.592, 0.909), 0.0047	0.798 (0.629, 1.013), 0.0640	0.788 (0.620, 1.002), 0.0520	
Q3(21–25)	0.605 (0.475, 0.770), < 0.0001	0.742 (0.558, 0.986), 0.0396	0.731 (0.548, 0.973), 0.0320	
Q4(26–38)	0.432 (0.338, 0.551), < 0.0001	0.528 (0.381, 0.733), 0.0001	0.546 (0.393, 0.760), 0.0003	
P for trend	0.956 (0.944, 0.968), < 0.0001	0.968 (0.951, 0.985), 0.0002	0.969 (0.952, 0.986), 0.0005	

In sensitivity analysis, OBS was converted from a continuous variable to a categorical variable (quartiles)

*OR* Odds ratio, *95% Cl* 95% confidence interval

^a^Model 1: No covariates were adjusted

^b^Model 2: Adjusted for age, race, education level, PIR and energy intake

^c^Model 3: Adjusted for age, race, education level, PIR, energy intake, hypertension and diabetes

The TOBS was subdivided into DOBS and LOBS to investigate their respective associations with RA. Similarly, both DOBS and LOBS were negatively correlated with RA when they were continuous variables (Tables 4 and 5). In Model 3, the OR (95%CI) of the third DOBS group was 0.677 (0.515 to 0.889), and the OR (95%CI) of the fourth DOBS group was 0.659 (0.470 to 0.925). The *P* for trend is 0.0071. Besides, the negative association between LOBS and RA was more pronounced, especially in the fourth LOBS group (OR = 0.573, 95%CI = 0.433 to 0.758). These results revealed that the antioxidant dietary and lifestyle may offer significant protection against RA.

**Table 4:** The relationship between DOBS and RA

	**OR (95% CI)**, ***P*-value**			
	**Model 1**^**a**^	**Model 2**^**b**^	**Model 3**^**c**^	
	RA			
DOBS	0.963 (0.951, 0.975), < 0.0001	0.975 (0.958, 0.992), 0.0047	0.975 (0.957, 0.992), 0.0047	
Q1(2–9)	Reference	Reference	Reference	
Q2(10–15)	0.747 (0.598, 0.933), 0.0102	0.831 (0.649, 1.064), 0.1417	0.816 (0.636, 1.047), 0.1104	
Q3(16–21)	0.584 (0.465, 0.734), < 0.0001	0.689 (0.525, 0.903), 0.0070	0.677 (0.515, 0.889), 0.0051	
Q4(22–31)	0.503 (0.395, 0.641), < 0.0001	0.650 (0.464, 0.910), 0.0122	0.659 (0.470, 0.925), 0.0158	
P for trend	0.961 (0.948, 0.974), < 0.0001	0.975 (0.957, 0.993), 0.0057	0.975 (0.957, 0.993), 0.0071	

In sensitivity analysis, OBS was converted from a continuous variable to a categorical variable (quartiles)

*OR* Odds ratio, *95% Cl* 95% confidence interval

^a^Model 1: No covariates were adjusted

^b^Model 2: Adjusted for age, race, education level, PIR and energy intake

^c^Model 3: Adjusted for age, race, education level, PIR, energy intake, hypertension and diabetes

**Table 5:** The relationship between LOBS and RA

	**OR (95% CI)**, ***P*-value**			
	**Model 1**^**a**^	**Model 2**^**b**^	**Model 3**^**c**^	
	RA			
LOBS	0.800 (0.758, 0.844), < 0.0001	0.838 (0.789, 0.891), < 0.0001	0.861 (0.809, 0.916), < 0.0001	
Q1(0–2)	Reference	Reference	Reference	
Q2(3–3)	0.984 (0.749, 1.292), 0.9063	0.932 (0.695, 1.249), 0.6366	0.936 (0.696, 1.257), 0.6598	
Q3(4–4)	0.766 (0.591, 0.993), 0.0442	0.797 (0.601, 1.055), 0.1129	0.804 (0.606, 1.068), 0.1324	
Q4(5–8)	0.443 (0.344, 0.570), < 0.0001	0.521 (0.395, 0.687), < 0.0001	0.573 (0.433, 0.758), 0.0001	
P for trend	0.798 (0.755, 0.843), < 0.0001	0.839 (0.789, 0.892), < 0.0001	0.862 (0.810, 0.917), < 0.0001	

In sensitivity analysis, OBS was converted from a continuous variable to a categorical variable (quartiles)

*OR* Odds ratio, *95% Cl* 95% confidence interval

^a^Model 1: No covariates were adjusted

^b^Model 2: Adjusted for age, race, education level, PIR and energy intake

^c^Model 3: Adjusted for age, race, education level, PIR, energy intake, hypertension and diabetes

Furthermore, to evaluate the influence of different OBS types, the Z-score standardization was applied. This approach assisted to measure the effect of each standard deviation increment of OBS on RA risk. In Model 3, the findings revealed a decrease in RA risk by 21.0%, 16.2%, and 20.9% for each standard deviation increase in TOBS, DOBS, and LOBS, respectively. These results indicated that, after adjusting for confounding factors, LOBS offered more protection against RA compared to DOBS, though it was marginally less effective than TOBS (Table 6).

**Table 6:** The relationship of three types of standardized OBS and RA

	**OR (95% CI)**, ***P*-value**			
	**Model 1**^**a**^	**Model 2**^**b**^	**Model 3**^**c**^	
	RA			
TOBS (Z-score)	0.724 (0.665, 0.788), < 0.0001	0.779 (0.691, 0.877), < 0.0001	0.790 (0.701, 0.891), 0.0001	
DOBS (Z-score)	0.771 (0.709, 0.839), < 0.0001	0.839 (0.743, 0.948), 0.0047	0.838 (0.742, 0.947), 0.0047	
LOBS (Z-score)	0.705 (0.648, 0.766), < 0.0001	0.759 (0.690, 0.835), < 0.0001	0.791 (0.717, 0.872), < 0.0001	

*OR* Odds ratio, *95% Cl*95% confidence interval

^a^Model 1: No covariates were adjusted

^b^Model 2: Adjusted for age, race, education level, PIR and energy intake

^c^Model 3: Adjusted for age, race, education level, PIR, energy intake, hypertension and diabetes

### Subgroup analyses

**Fig. 2:**
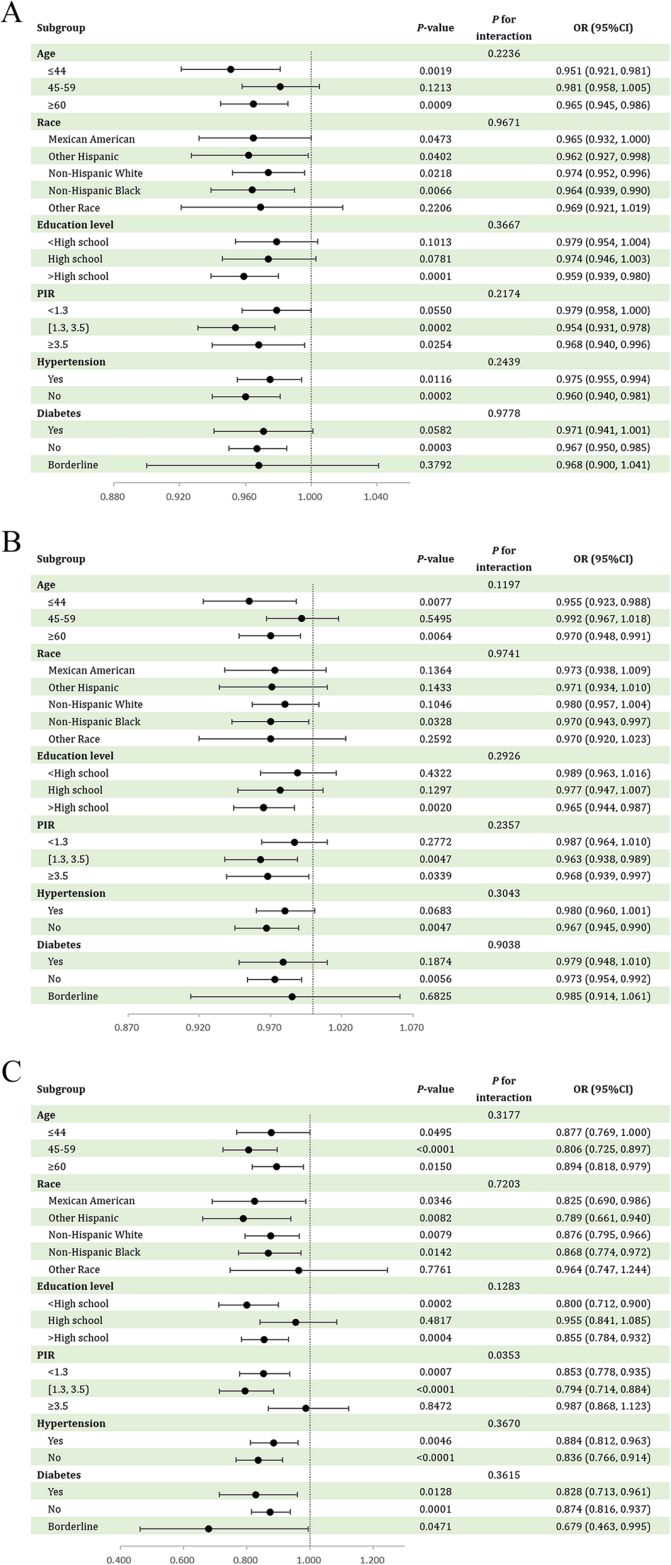
** A** Forest plot of subgroup analyses for the associations between TOBS and RA. **B **Forest plot of subgroup analyses for the associations between DOBS and RA. **C** Forest plot of subgroup analyses for the associations between LOBS and RA

Subgroup analyses and interaction tests were used to assess the associations between the three types of OBS and RA (Fig. 2A and C). The results showed that age, race, education level, PIR, hypertension and diabetes had no significant modification effect on the inverse link between TOBS, DOBS and RA (*P* for interaction > 0.05). Nevertheless, PIR exhibited a modifying effect on the relationship between LOBS and RA (*P* for interaction = 0.0353), as shown by the significant protective effect of LOBS against RA in low and middle-income, but not high-income populations.

### Smooth curve fitting and threshold effect analysis

**Fig. 3:**
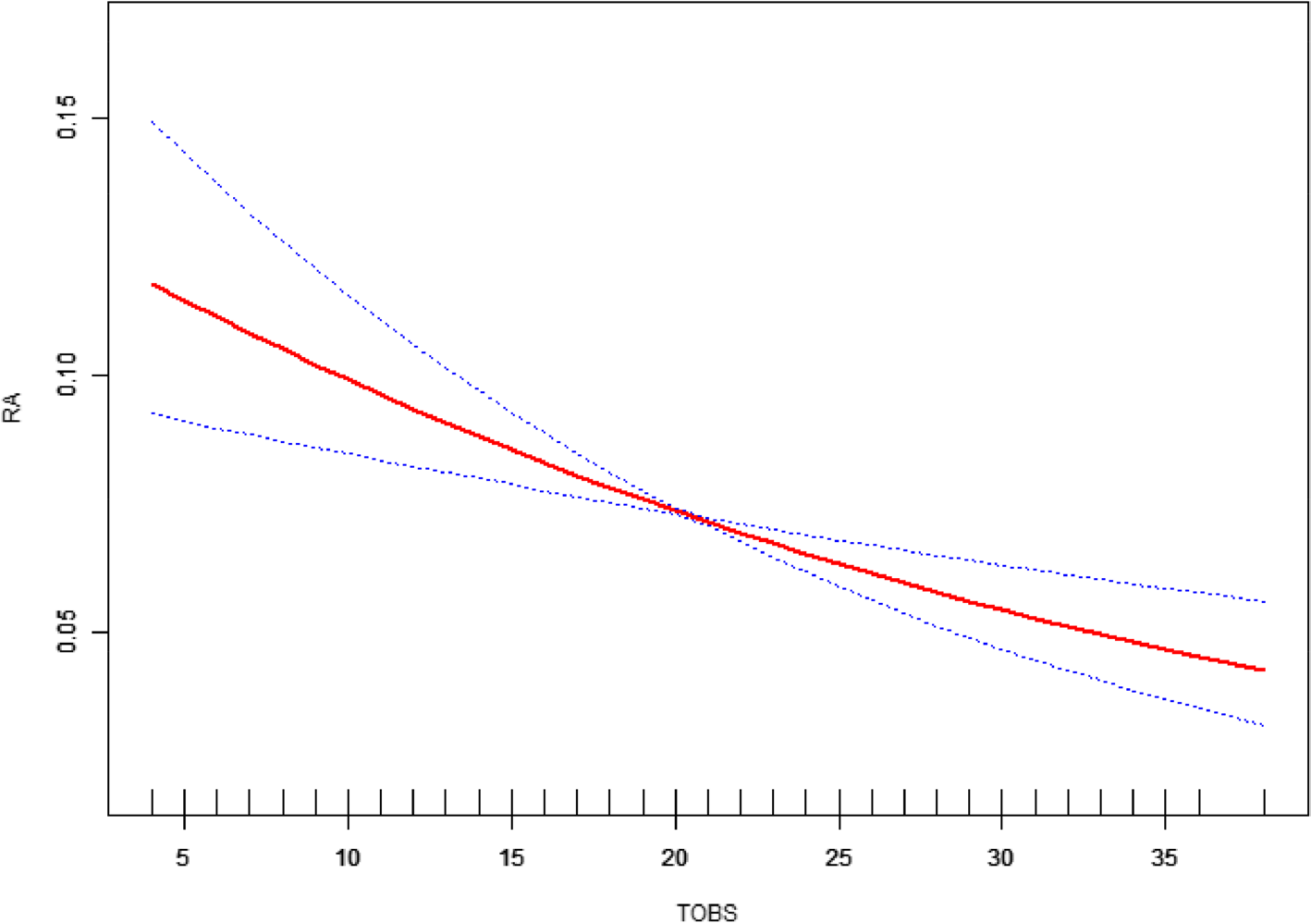
Smooth curve fitting for TOBS and RA. Adjusted for age, race, education level, PIR, energy intake, hypertension and diabetes

A smooth curve fitting model was established and threshold effects were calculated to ascertain the linearity of the relationship between TOBS and RA (Fig. 3). After adjusting all covariates, two relationship models of TOBS and RA were created through threshold effect analysis (Table 7). The *P*-value of the log-likelihood ratio test was 0.163, suggesting that linear effect model was supposed to be selected to confirm the negative correlation between TOBS and RA (OR = 0.968, 95%CI = 0.952 to 0.984), which was consistent with multivariate logistic regression and smooth curve fitting results.

**Table 7:** Threshold effect analysis of TOBS and RA

RA	Adjusted OR (95% CI), *P* -value
TOBS	
Linear regression model	0.968 (0.952, 0.984), 0.0001
Two-segment piecewise linear regression model	
Inflection point	12
TOBS < Inflection point	0.918 (0.852, 0.990), 0.0262
TOBS > Inflection point	0.975 (0.956, 0.994), 0.0099
Log-likelihood ratio	0.163

Adjusted for age, race, education level, PIR, energy intake, hypertension and diabetes

## Discussion

Since RA is more common in women, we conducted this cross-sectional study using 597 RA patients and 7622 participants without RA from the NHANES database for this gender. This study pointed out that the higher the OBS of the female population, the lower the prevalence of RA, signifying a negative correlation between all three kinds of OBS and RA. In a fully adjusted model that accounted for all confounders, for each unit increase, the risk of RA was decreased by 3.2% in TOBS, 2.5% in DOBS, and 13.9% in LOBS. In addition, subgroup analyses results suggested the subgroups exhibited no significant modification effect of the negative correlation between OBS and RA, except for the PIR subgroup in LOBS. Moreover, a smooth curve fitting model was established and further verified the negative linear relationship between OBS and RA. Collectively, the results indicated that OBS, which represents the bodily oxidative stress state, is a simple and effective tool for the epidemiological study of RA.

RA is a complex disease characterized by diverse progression rates and varied treatment responses. The present-day assessment of its activity and severity is rooted in a blend of clinical examinations and laboratory analyses, which guide treatment strategies. The employment of biomarkers has been eyed as a potential avenue for delivering a more precise, objective evaluation of disease states. The academic arena has seen a burgeoning interest in biomarkers pertinent to arthritis risk, development, and prognosis. Slama et al. [32] affirmed the efficacy of both the Clinical Disease Activity Index and the Simplified Disease Activity Index (SDAI) in appraising disease activity among RA patients within the Moroccan demographic. Diving into a novel approach, González-Álvaro et al. [33] devised a straightforward and easily accessible index, termed Hospital Universitario La Princesa Index (HUPI), to measure RA disease activity. They advocate that HUPI eclipses SDAI when it comes to distinguishing between low-to-moderate disease activity, and it surpasses DAS28 in pinpointing clinical remission. On a different note, Xu et al. [34] orchestrated a multicentric retrospective exploration, unveiling that when pitted against the neutrophil/lymphocyte ratio, monocyte/lymphocyte ratio, and platelet/lymphocyte ratio, the Systemic Inflammatory Response Index emerges as a promising new biomarker. This index might ease the diagnostic journey and mirror disease activity in RA patients, with the potential to predict the emergence of RA-associated interstitial lung disease and tumors. Moreover, our prior investigation discerned a non-linear positive correlation between the Triglyceride Glucose Index, a biomarker indicative of insulin resistance, and arthritis [35]. Yet, the scholarly domain hasn’t delved into exploring the potential interplay between OBS, a novel accessible biomarker for oxidative stress, and RA.

To our knowledge, this investigation pioneers a population-based study aimed at discerning the relationship between OBS and RA. Historically, a multitude of studies has explored the nexus between OBS and diverse ailments, employing a spectrum of epidemiological approaches across varied demographic cohorts. A comprehensive cross-sectional study by Liu et al. [36] encompassing 18,716 American participants, unearthed a significant and stable negative non-linear relationship between OBS, DOBS, LOBS, and depression (*P* for non-linear < 0.05), with a more pronounced correlation in women. Lei et al. [37] surveyed 6,300 individuals, unveiling that OBS bore a negative correlation with sleep disturbance (OR = 0.97; 95%CI = 0.94 to 0.99), and a positive one with sleep duration (mean difference = 0.02, 95%CI = 0.01 to 0.03), particularly among women below 50 (*P* < 0.001). Further afield, Yeo et al. [38] studied 5,807 Korean subjects, finding that higher OBS corresponded with significantly smaller neck circumferences, indicative of a possible oxidative imbalance mirrored in central obesity. Song et al. [39] investigated 1,745 American seniors, and discerned a positive correlation between OBS and cognitive function, with each one-unit increase in OBS associated with a 0.03 point increase in global cognitive function scores in a fully adjusted model, and that albumin, uric acid, and serum 25 (OH)-D concentrations potentially mediated this association. Shifting the focus to bone health, Shahriarpour et al. [40] conducted a study on 151 postmenopausal Iranian women aged between 50 and 85 years, revealing that a higher OBS, signifying a dominance of antioxidant exposures, was associated with a reduced risk of lumbar spine osteoporosis. In a similar vein, Cho et al. [41] determined that a higher OBS significantly correlated with a lower risk of non-alcoholic fatty liver disease incidence in 10,030 Korean middle-aged and elderly individuals. Prospective cohort studies by Son et al. [42] and Lee et al. [43] respectively unearthed that higher OBS was linked with a lower risk of chronic kidney disease [hazard ratio (HR) = 0.94, 95%CI = 0.91 to 0.97) and new-onset hypertension(HR = 0.94, 95%CI = 0.92 to 0.97]. Liu et al. [44] found that TOBS and DOBS levels were positively correlated with vascular endothelial function, revealing another link between OBS and cardiovascular disease. Qu et al. [45] illustrated a negative linear association between OBS and periodontitis among US adults, and the risk of periodontitis was reduced by 11% for every 1 unit increase in OBS, proposing OBS as a viable biomarker for periodontitis evaluation. The accruing body of evidence underscores the potential of OBS in evaluating a gamut of diseases, laying a robust foundation for our trailblazing study aimed at elucidating the relationship between OBS and RA.

Beyond identifying a robust association between OBS and RA, an intriguing aspect of our analysis revealed that PIR exerted a modulatory effect on the relationship between LOBS and RA. Specifically, this interaction emerged because the association between LOBS and RA did not hold significance within the cohort with high PIR. This observation aligns with previous research investigating lifestyle factors across different income brackets. For instance, Birch et al. [46] highlighted that higher income brackets might mitigate the adverse health effects of smoking, indicating that economic advantage could lessen vulnerability to such lifestyle risks. While Lewer et al. [47] found a propensity for excessive alcohol consumption in lower socio-economic groups, correlating with a higher susceptibility to alcohol-related health issues. Moreover, a prospective study confirmed the link between higher income levels and healthier lifestyle choices, ultimately leading to a diminished risk of negative health outcomes [48]. Consequently, it is plausible that populations with higher incomes possess greater means and opportunities for maintaining healthful lifestyles, which not only improves baseline health but also decreases RA risk. This scenario likely accounts for the diluted influence of antioxidant-rich lifestyles on RA among those with high PIR, manifesting as a non-significant LOBS-RA association in these groups.

The underlying mechanisms driving the association between OBS and RA remain unclear. The link between oxidative stress and RA is intricate and multi-faceted. The following points can help illuminate the latent mechanisms between oxidative stress and RA. Firstly, under normal circumstances, the body also naturally produces free radicals as well as ROS. Oxidative stress arises with an excess of free radicals or ROS, which can attack cellular lipids, proteins and DNA, conducing to cellular damage and inflammatory responses. In RA patients, joint synovial cells and chondrocytes may produce excessive free radicals, resulting in tissue destruction and inflammation. An observational study conducted by Ogul et al. [49] suggested that malondialdehyde, a strong marker of free oxygen radicals, was notably elevated in RA patients. Khojah H. M. et al. [50] found that reactive oxygen species (including superoxide anion, hydroxyl radical, hydrogen peroxide, and peroxyl radical) and reactive nitrogen (including nitric oxide and peroxynitrites) levels of RA patients were significantly higher than those in the control group. In turn, the body has an antioxidant defense mechanism to neutralize free radicals and ROS. However, these antioxidant defense mechanisms can be compromised in RA patients, leading to intensified oxidative stress. Catalase, a crucial element of the human antioxidant defense mechanism that protects cells against ROS damage, was reported to be reduced in erythrocytes from RA patients [51]. Furthermore, decreased levels of glutathione defense systems, including glutathione and glutathione peroxidase, are closely associated with RA [52]. Secondly, there exists a remarkable association between oxidative stress and inflammation. The accumulation of free radicals, ROS, and reactive nitrogen species can activate and regulate a series of inflammation signal transduction pathways, in particular the nuclear factor kappa-B (NF-κB) [53, 54] and mitogen activated protein kinase (MAPK) [55] signaling pathways. For example, on the one hand, ROS can induce the activation of NLRP3 inflammasome, which interacts with NF-κB signal [56], and on the other hand, it mediates the activation of MAPK pathway [57], thereby promoting the generation and secretion of inflammatory cytokines and mediators, exacerbating the inflammatory response and joint damage of RA. Thirdly, excessive oxidative stress may not only activate cell death signals, triggering chondrocyte injury, further aggravating joint tissue destruction [58], but also affect cell functions by changing cell signal transduction pathways, including cell proliferation, differentiation, and migration [59]. All these may affect the progress of RA. Fourthly, studies have shown that strong antioxidants such as vitamin C and vitamin E may help reduce oxidative stress and thus combat the symptoms of RA [60]. These antioxidants neutralize free radicals and reduce the inflammatory response, probably benefiting from alleviating symptoms of RA.

### Strengths and limitations

This study bolstered the evidence for the negative linear correlation between OBS and RA, enriching the existing literature. And generalizability of results was enhanced by the use of this comprehensive, representative cohort from the NHANES database. In addition, this study boosts the understanding of OBS, which is a potent marker to accurately locate RA-susceptible individuals and highlights the consequence of oxidative stress in the pathogenesis of RA.

Nevertheless, there are certain limitations to this study. It is important to note that due to the constraints of NHANES database, the diagnosis of RA in this study was obtained through participant questionnaires. This method may introduce recall bias, potentially affecting the accuracy of the diagnoses. Besides, the cross-sectional design fails to identify a temporal connection between OBS and RA. Prospective cohort studies are strongly required to further delineate the causal relationship between OBS and RA. Additionally, although many covariates were included in this study to reduce the influence of confounders, numerous contributing factors were still not considered, which may have an impact on the association between OBS and RA. Furthermore, this study only involved population in the United States, and data from public databases may have resulted in subject selection bias. Moreover, the characteristics of RA, such as chronic pain, limited mobility, high disability rate, and chronic disease course, can severely affect patients’ life, work, and education, and increase the financial burden of patients, which contribute to the low age, high disability rate, as well as the low income of patients. These factors may also promote selection bias. And future studies should include more female population with backgrounds of other genetic and environmental factors to increase the generalizability of the results.

## Conclusion

In conclusion, OBS serves as a robust indicator of the body’s oxidative stress level. Our study revealed that TOBS, as well as DOBS and LOBS, all negatively correlated with RA in women. Moreover, it is meaningful to develop prevention measures for RA based on OBS. Advocating antioxidant dietary patterns and an antioxidant healthy lifestyle can enhance OBS and may protect the female population from RA. In contrast, pro-oxidant factors such as excessive fat intake, obesity, and smoking reduce OBS, which may accelerate RA development. More studies on OBS and RA in the future will assist in better illuminating their correlation.

### Supplementary Information

**Supplementary Material 1.**

**Supplementary Material 2.**

## Data Availability

Publicly available datasets were analyzed in this study. This data can be found here: https://wwwn.cdc.gov/nchs/nhanes. In addition, the authors uploaded the original data as a supplementary file, as shown in Supplementary Material 2.

## Abbreviations

BMI: Body mass index
CI: Confidence intervals
DOBS: Dietary OBS
HR: Hazard ratio
HUPI: Hospital Universitario La Princesa Index
LOBS: Lifestyle OBS
MAPK: Mitogen activated protein kinase
MET: Metabolic equivalent
NF-κB: Nuclear factor kappa-B
NHANES: National Health and Nutrition Examination Survey
OBS: Oxidative balance score
OR: Odds ratio
PIR: Poverty income ratio
RA: Rheumatoid arthritis
ROS: Reactive oxygen species
SDAI: Simplified Disease Activity Index
SII: Systemic immune inflammation index
TOBS: Total OBS
