# Supplementary material for: Association between oxidative balance score and rheumatoid arthritis in female: a cross-sectional study
Source: BMC Womens Health. 2024 Apr 6;24:225. doi: 10.1186/s12905-024-03066-3 (PMC10998364; doi:10.1186/s12905-024-03066-3)
Supplement: Supplementary file 1 — Supplementary Material 1. [file 12905_2024_3066_MOESM1_ESM.docx]

**Association between oxidative balance score and** **rheumatoid arthritis in female: a cross-sectional study**

# Supplementary Material

| Supplementary Table 1: The original scores of 20 items of TOBS for RA and Non-RA | | | |
| --- | --- | --- | --- |
| OBS components | Non-RA | RA | *P* value |
| Dietary fiber | 1.02 ± 0.81 | 0.84 ± 0.82 | <0.001 |
| Carotene | 1.01 ± 0.81 | 0.93 ± 0.84 | 0.028 |
| Riboflavin | 1.01 ± 0.82 | 0.88 ± 0.82 | <0.001 |
| Niacin | 1.01 ± 0.82 | 0.83 ± 0.81 | <0.001 |
| Total folate | 1.02 ± 0.82 | 0.84 ± 0.81 | <0.001 |
| Vitamin B6 | 1.01 ± 0.82 | 0.85 ± 0.82 | <0.001 |
| Vitamin B12 | 1.01 ± 0.82 | 0.91 ± 0.81 | 0.008 |
| Vitamin C | 1.00 ± 0.81 | 0.94 ± 0.84 | 0.076 |
| Vitamin E | 1.01 ± 0.82 | 0.86 ± 0.80 | <0.001 |
| Calcium | 1.01 ± 0.82 | 0.87 ± 0.83 | <0.001 |
| Magnesium | 1.01 ± 0.82 | 0.84 ± 0.81 | <0.001 |
| Zinc | 1.01 ± 0.82 | 0.83 ± 0.81 | <0.001 |
| Copper | 1.01 ± 0.82 | 0.88 ± 0.81 | <0.001 |
| Selenium | 1.01 ± 0.82 | 0.84 ± 0.80 | <0.001 |
| Iron | 0.99 ± 0.82 | 1.10 ± 0.82 | 0.001 |
| Total fat | 0.99 ± 0.82 | 1.13 ± 0.80 | <0.001 |
| Physical activity | 0.85 ± 0.80 | 0.63 ± 0.80 | <0.001 |
| Alcohol | 1.64 ± 0.67 | 1.76 ± 0.58 | <0.001 |
| Body mass index | 0.95 ± 0.84 | 0.66 ± 0.79 | <0.001 |
| Cotinine | 1.00 ± 0.81 | 0.84 ± 0.79 | <0.001 |
